# TRII: A Probabilistic Scoring of *Drosophila melanogaster* Translation Initiation Sites

**DOI:** 10.1155/2010/814127

**Published:** 2010-10-18

**Authors:** Michael P Weir, Michael D Rice

**Affiliations:** 1 Department of Biology , Wesleyan University, Middletown, CT 06459, USA; 2 Department of Mathematics and Computer Science, Wesleyan University , Middletown, CT 06459, USA

## Abstract

Relative individual information is a measurement that scores the quality of DNA- and RNA-binding sites for biological machines. The development of analytical approaches to increase the power of this scoring method will improve its utility in evaluating the functions of motifs. In this study, the scoring method was applied to potential translation initiation sites in Drosophila to compute Translation Relative Individual Information (TRII) scores. The weight matrix at the core of the scoring method was optimized based on high-confidence translation initiation sites identified by using a progressive partitioning approach. Comparing the distributions of TRII scores for sites of interest with those for high-confidence translation initiation sites and random sequences provides a new methodology for assessing the quality of translation initiation sites. The optimized weight matrices can also be used to describe the consensus at translation initiation sites, providing a quantitative measure of preferred and avoided nucleotides at each position.

## 1. Introduction

Understanding how biological machines work in the context of genomes, transcriptomes, and proteomes requires appropriate languages and representations for successful modeling of their biological processes. Information theory provides one of the foundations for this goal and underlies sequence motif-finding algorithms such as *MEME* [1]. For example, information theory gives us powerful ways to analyze and score sequence motifs in RNAs that are targeted by biological machines such as the spliceosome or ribosome [2–4]. The approach reveals, for each nucleotide position in the motif, which nucleotide choices are preferred and which are avoided. For any single RNA sequence, the collective deviations from the preferred nucleotides must be sufficiently small for the machine to successfully function on that RNA.

In this study, several analytical approaches are integrated to increase the power of these scoring methods using Drosophila translation initiation sites as a model setting. As an introduction, we describe first the information theoretic basis for these scoring methods. Motifs of functional importance can be quantitatively assessed through their sequence conservation, measured as information content in sets of aligned sequences [2, 5, 6]. The information at each nucleotide position  for a set of  aligned RNA sequences is defined by the expression(1)

The summation represents the uncertainty based on the frequencies of occurrence  of the nucleotides  at position . The sampling correction factor  depends on  and decreases toward 0 as the value of  increases [3]. 

It is sometimes important to take into account nonrandom background nucleotide frequencies. For example, the mean frequencies of each nucleotide in Drosophila cDNAs deviate significantly from 0.25 [3], and this fact may influence how spliceosomes or ribosomes perceive RNA molecules. The *relative information* (often called relative entropy) at each nucleotide position  is defined by the expression(2)

where  is the background frequency of nucleotide  in a selected set of sequences.

The information values defined above are based on *groups* of aligned sequences. The theory can be extended to allow assessment of *individual* sequences. Measurement of individual information allows scoring of how well an individual sequence conforms to a conserved motif [7]. For example, it has been used to score conserved motifs such as splice sites [3]. Individual information is defined with respect to a reference set  of aligned sequences as follows. Assume that  consists of  aligned sequences, each of length . Suppose that  denotes the nucleotides in a test sequence . Then, the*individual information* of  is defined by(3)

where  denotes the frequency of occurrence of nucleotide  at position  in the set , and  denotes the sampling correction factor discussed above. In essence, the reference set  is used to create a weight matrix of values  which are used to calculate the individual information score based on which nucleotide  is present at each position  in the test sequence . The more representative the reference sequences used to construct the weight matrix, the better the dynamic range of the individual information scoring system: sequences with a good match to a motif will have higher scores, and sequences with poorer matches will have lower scores (see discussion of matrix optimization below).

Nonrandom background nucleotide frequencies can be taken into account using *relative individual information* (sometimes called "individual relative entropy") which is defined as follows: (4)

where  is the background frequency of nucleotide . For example, when relative individual information is used to score splice sites [3], background nucleotide frequencies based on the full set of cDNAs were used. 

Relative individual information scoring of individual DNA and RNA sequences has been discussed previously [7], and forms the basis for motif finding algorithms such as *MEME* [1] which are based on Markov models that encapsulate the notion of individual information. In this study, we developed methods to use relative individual information to score translation initiation sites using Drosophila as a model system. When applied to translation initiation, we refer to relative individual information scores as TRII scores (Translation Relative Individual Information). As presented below, the ability to score individual sequences presents an opportunity to analyze *distributions* of TRII scores for sets of sequences of interest. By appropriate choices of control test TRII score distributions, this approach allows one to interpret score distributions for sites of interest in a probabilistic manner. Analysis of score distributions provides insights into translation initiation: potential initiation sites with TRII scores that resemble high-confidence start sites can be considered likely initiation sites whereas sites similar to random sequences are likely to be weak or nonfunctional for translation initiation. We also discuss how the methods described in this paper can be applied to the initiation context scoring method of Miyasaka [8] which has been used, for example, to predict and score translation initiation sites in a recent ribosome profiling study based on deep sequence analysis in yeast [9]. In contrast to TRII scoring, which measures deviations from background frequencies at each nucleotide position (4), the Miyasaka method is based on deviations from the preferred nucleotide at each position.

## 2. Results and Discussion

### 2.1. Identification of High-Confidence Translation Initiation Sites

An initial goal of this analysis was to define sets of high-confidence translation start sites whose TRII score distributions could be used as standards for analysis of TRII score distributions of other test sets. Previous studies have tended to rely on "curated" gene sets to define training sets of high-confidence translation initiation sites. Instead, we developed a bioinformatics approach to identify large sets of initiation sites in which we could have high confidence.

**Figure 1:**
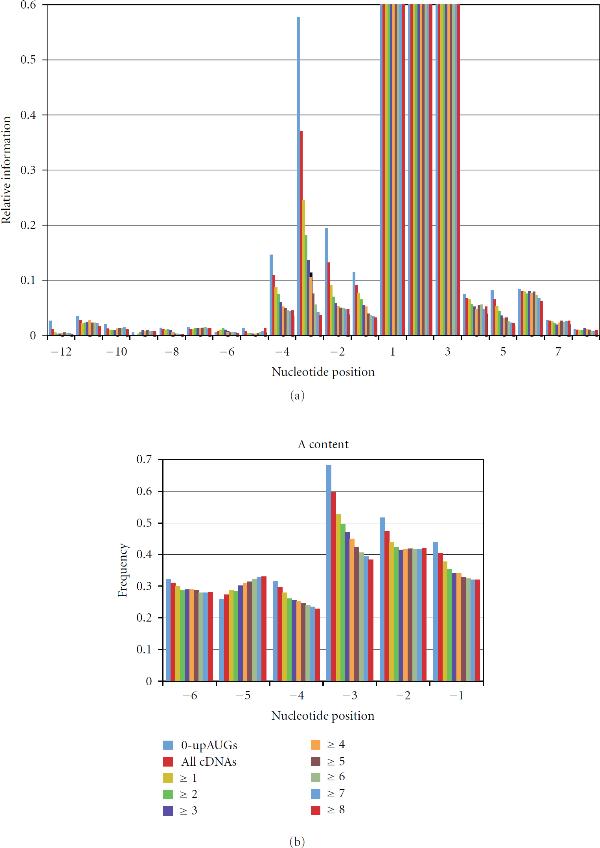
**Progressive partitioning of annotated start sites based on number of upstream AUG codons**. Nucleotide position −3 exemplifies the elevation of relative information (a) and A content (b) with 0-upAUGs and the progressive decrease with higher numbers of upAUGs (≥1 through ≥8). Nucleotide positions are numbered relative to the AUG which have relative information of 1.7, 2.0 and 2.2 bits, respectively, (not shown). The following background frequencies in the 5′UTRs of 8,607 cDNAs were used in all figures: , , , and .

In previous studies [3, 4], we showed that progressive partitioning of large genomic datasets can identify special subsets of sequences with stronger conservation of sequence motifs. For example, splice sites adjacent to longer introns or exons have particularly high sequence conservation [3]. In the current analysis, we studied a set of annotated translation start sites (annAUGs) in 8,607 Drosophila cDNAs that were sequenced by the Berkeley Drosophila Genome Project [10–12]. Partitioning this set of cDNAs based on the number of upstream AUGs (upAUGs) present in the annotated UTR revealed a striking result (Figure 1). Relative information levels near annAUGs are much higher in subsets of cDNAs with fewer upAUGs. This is particularly pronounced, for example, at nucleotide position −3 (the 3rd nt upstream of the AUG found at positions 1, 2 and 3; Figure 1). Consistent with this result, the presence of upAUGs in 5^'^UTRs has been associated previously with weak contexts of translation start codons in several organisms [13].

We hypothesized that the depressed relative information levels at annAUGs associated with upAUGs might be explained by the presence of annAUGs that are weak or nonfunctional translation initiation sites. For example, weak or nonfunctional annAUG sites might be expected if there is translation initiation at upAUGs followed by translation reinitiation [14–16] at annAUGs or downstream AUGs. To investigate this further, the distributions of relative individual information scores were examined for subsets of cDNAs with different numbers of upAUGs. We assessed whether the subsets of cDNAs with different numbers of upAUGs were essentially a mixture of two classes of annAUGs: (i) higher-scoring, likely functional translation start sites and (ii) lower-scoring, weak, or nonfunctional start sites. 

The translation relative individual information (TRII) scores were calculated using a reference set  which we define as the set of cDNAs whose 5^'^UTRs contain at least 200 nucleotides (denoted 5^'^UTR ≥ 200; see Supplementary Table 6 for summary of sequence sets used in this study available online at: doi:10.1155/2010/814127). Because ribosomes are hypothesized to scan 5^'^UTRs to identify translation initiation sites, we used the nucleotide frequencies in the 5^'^UTRs of a set of 8,607 cDNAs as background frequencies. The weight matrix is based on these background frequencies and nucleotide positions −20 to 20 relative to the annAUGs in . This range of positions is used throughout the paper to define weight matrices and to score test sequences. 

**Figure 2:**
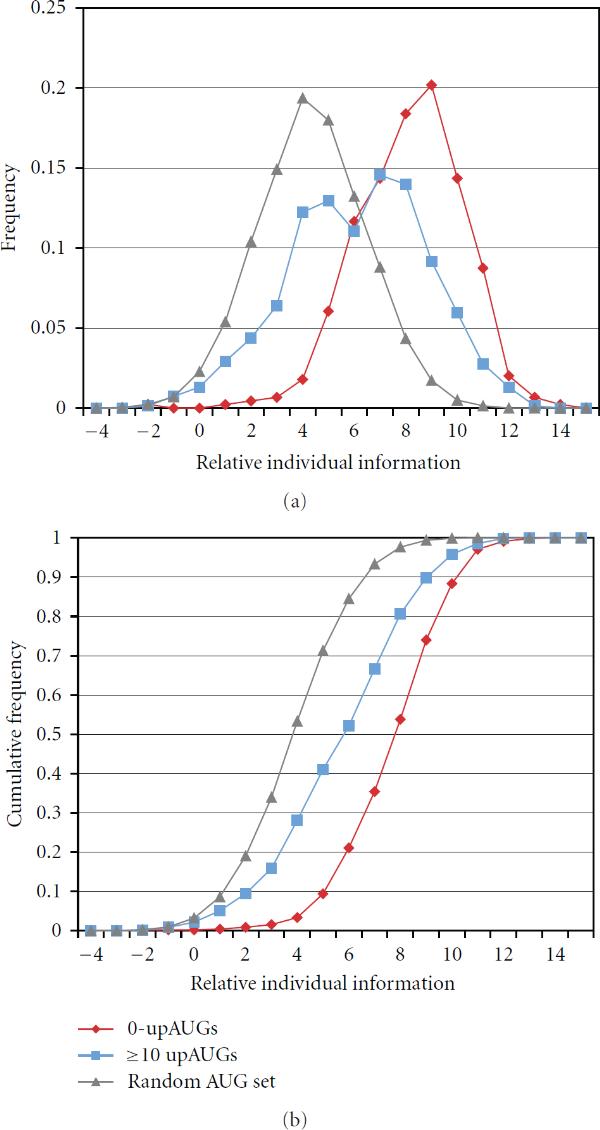
**Relative individual information score distributions (a) and corresponding cumulative distributions (b)**. The annAUGs of the full set of cDNAs with 5^'^UTR ≥ 200 were used as a reference set to construct the weight matrix for nucleotide positions −20 to 20. Three test sets were compared: (i) 0upAUGs, 5^'^UTR ≥ 200 (red); (ii) 687 cDNAs with at least 10 upAUGs, 5^'^UTR ≥ 200 (blue); (iii) AUGs surrounded with random sequences conforming to the 5^'^UTR background frequencies (grey). In this example, the reference set  includes the 0-upAUG test set (red); however, the use of nonoverlapping reference and test sets is preferred (see text).

We compared a control test set of cDNAs with no upAUGs (0-upAUGs with 5^'^UTR ≥ 200) with a series of test sets of cDNAs with increasing numbers of upAUGs (and 5^'^UTR ≥ 200). To represent weak or nonfunctional annAUGs, we generated the set  consisting of 5000 sequences with AUGs surrounded by random sequences (at positions −20 to −1 and 4 to 20) conforming to the 5^'^UTR background nucleotide frequencies. Figure 2 illustrates, as an example, the distribution of scores for the subset of 687 cDNAs with ≥10 upAUGs. Its distribution is slightly more spread out ( bits) compared to either the distributions of scores of the 0-upAUG test set ( bits) or the random sequence set ( bits). 

The shape of the score distribution for the test set with ≥10 upAUGs suggests that the scores may represent a combination of two overlapping distributions, a lower-scoring set of weak or nonfunctional annAUGs (with scores similar to the random AUG set), and a higher-scoring set of likely functional annAUGs (represented by the 0-upAUG set). For the test set with ≥10 upAUGs, a large fraction (approximately one-half) of the annAUGs appears to be low scoring and possibly nonfunctional (see Figure 2(a)). As expected from Figure 1, analysis of the score distributions for test sets with progressively more upAUGs shows progressively larger fractions of low-scoring sites (Table 1). 

**Table 1:** UpAUG Analysis

Number of upAUGs	Number of cDNAs	Random curve (%)	0-upAUG curve (%)
1	502	6	94
2 or 3	812	13	87
4 or 5	695	24	76
6 to 9	487	31	69
≥10	687	51	49

The annAUG TRII score distributions were computed for sets of cDNAs with different numbers of upAUGs (see, e.g., Figure 2).

Estimated fraction of cDNAs with random sequences in annAUG region, computed using reconstruction of TRII score distributions (see Methods).

The relative individual information distribution for the 0-upAUG set suggests it has the least contamination with weak or nonfunctional annAUGs, compared to sets of cDNAs with upAUGs in their UTRs (Figure 2 and data not shown). We conclude that identification of 0-upAUG sets provides a convenient informatics-based method for computing sets of high-confidence translation initiation sites.

### 2.2. Optimizing the Choice of the Reference Set

These sets of high-confidence translation initiation sites were used to improve the TRII scoring approach in two ways: (i) to modify the weight matrices that underpin the TRII scoring method, and (ii) to provide control test score distributions for assessment of scores. We first discuss optimization of the weight matrix. Up to this point, we have used  the full set of cDNAs with 5^'^UTR ≥ 200 as a reference set to construct the weight matrix for computing relative individual information scores. Because the 0-upAUG set consisting of 446 sequences appears to have least contamination with weak or nonfunctional start annAUGs, we explored using it instead as an optimized high-confidence reference set . Henceforth, we reserve the notation  and  for 0-upAUG sets with 5^'^UTRs ≥ 200 or between 100 and 199, respectively. 

**Figure 3:**
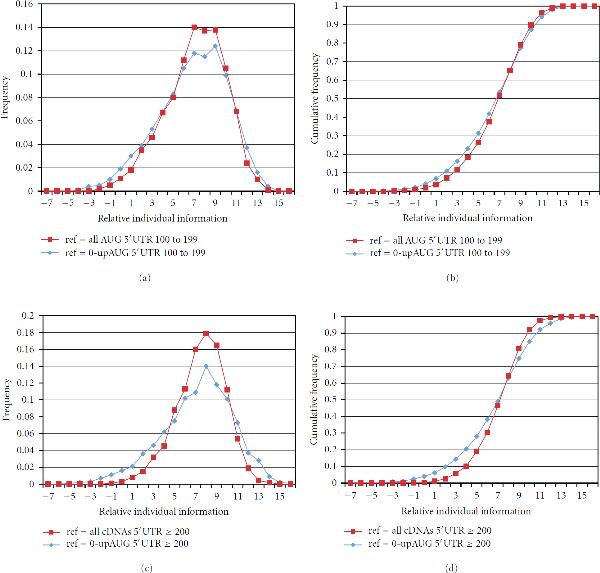
**Choice of weight matrix reference set**. (a, b) The test set of 3470 annAUGs with 5^'^UTR ≥ 200 is displayed using two different reference sets to construct weight matrices: (i)  (blue) and (ii) all cDNAs with 5^'^UTRs 100 to 199 (red). (c, d) Equivalent analysis using a test set of 1922 annAUGs (5^'^UTRs 100 to 199) and the reference sets (i)  (blue) and (ii) all cDNAs with 5′UTR ≥ 200 (red). In both analyses, using the 0-upAUG reference set expands the range of relative individual information scores. (a, c) TRII score distributions. (b, d) corresponding cumulative distributions.

We observed that using 0-upAUG reference sets gives a greater spread of relative individual information values—a higher "dynamic range" of scores—compared to using the set of all annAUGs as a reference set (Figure 3). The entries in the 0-upAUG weight matrix are of greater magnitude; hence, low-scoring annAUGs score lower because their inappropriate nucleotide choices lead to more pronounced negative weight contributions to the score, and high-scoring annAUGs score higher because the weights are greater for preferred nucleotides (compare weight matrices in Supplementary Tables 3, 4 and 5). This suggests that either one of the two purer 0-upAUG reference sets  or  is preferable for constructing the weight matrix. 

The use of 0-upAUG reference sets is supported by our testing of the TRII score method in budding yeast (Supplementary Figures 5 and 6). Protein expression and ribosome densities have been measured for most yeast genes [17, 18]. For highly expressed genes, we observed a correlation between TRII scores and protein expression levels or ribosome densities, and these correlations were stronger when a 0-upAUG reference set is used to compute the TRII scores (see Supplementary Material S.6).

**Figure 4:**
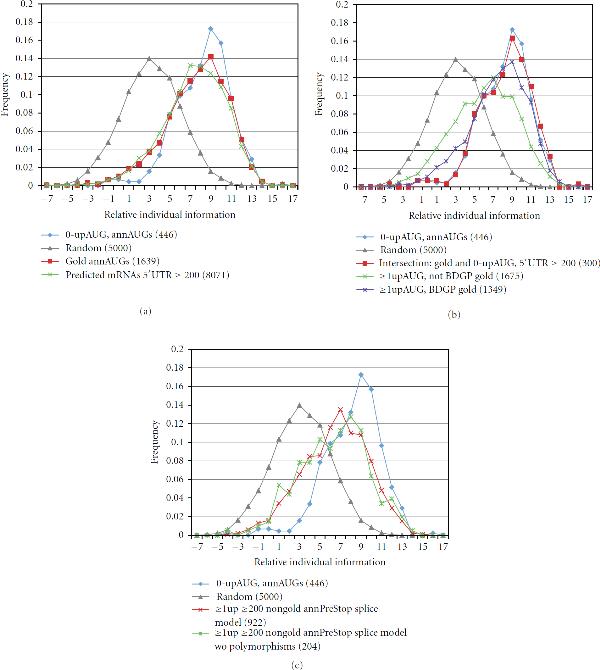
**TRII score distributions using  as a reference set for the weight matrix**. (a) The annAUGs of the set of 1,649 gold-set cDNAs with 5′UTR ≥ 200 (red) have a similar TRII score distribution to the set of 8,071 predicted mRNAs in *Release 5.9* with 5′UTR ≥ 200 (green). Both of these are similar to the distribution for 0-upAUG cDNAs (; blue), validating  as a control test distribution. (b) The set  (blue) and the subset of 300 gold-set 0-upAUG cDNAs (red) have similar score distributions. However, the set of 1,675 nongold-set cDNAs with ≥1 upAUG (green) has a higher fraction of low-scoring cDNAs than the 1,349 gold-set cDNAs with ≥1 upAUG (purple) (, chi-square goodness of fit). Given that nongold cDNAs represent mRNAs not in the predicted transcriptome, this suggests that that algorithms used to predict the Drosophila transcriptome were conservative and failed to predict significant numbers of experimentally observed transcripts including mRNAs with upAUGs and low-scoring annAUGs. (c) The conclusion in (b) is supported by analysis of subsets of nongold cDNAs (≥1 upAUG) that were aligned with genomic DNA using splice site-scanning algorithms [3, 4], either allowing single-nucleotide polymorphisms (992 cDNAs; red) or not (204 cDNAs; green). The distributions for both subsets and the full set (green curve in (b)) are similar. Note that the cDNAs in both subsets all have a stop codon upstream and in-frame with the annAUG. Moreover, premature termination by reverse transcriptase may apply to only a small fraction of these cDNAs: for 13 of the 204 cDNAs (green curve), the 5′ end of the cDNA matches an internal segment of a *Release 5.9* predicted transcript, and the cDNA sequence lies downstream of the predicted transcript's start codon.

**Figure 5:**
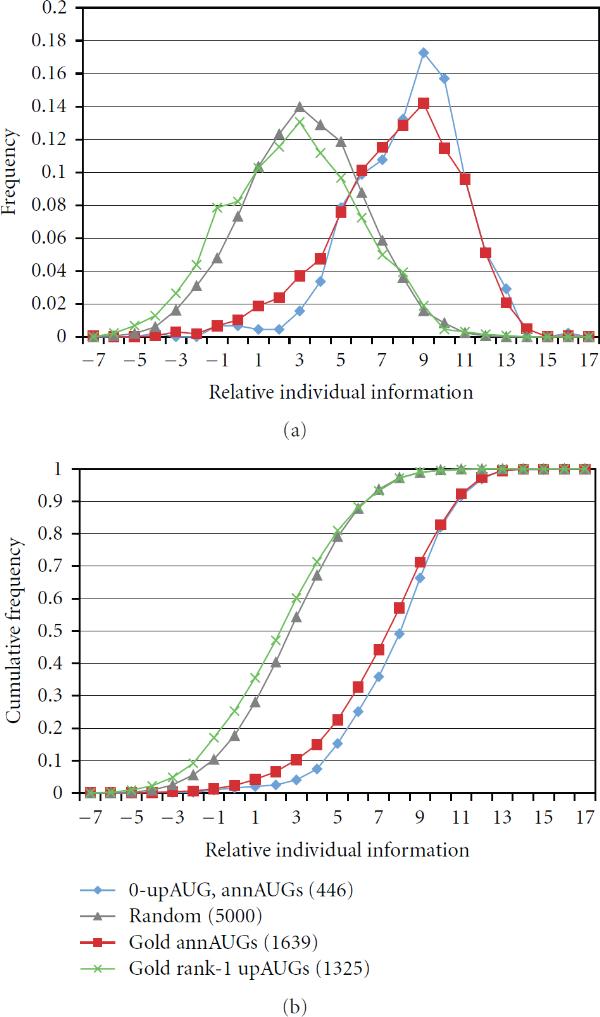
**UpAUGs have poor TRII scores**. The score distributions for the upAUG sequences of 1325 gold set cDNAs and the control set  are similar. The first AUG upstream of the annAUG in each cDNA was chosen for analysis.

**Figure 6:**
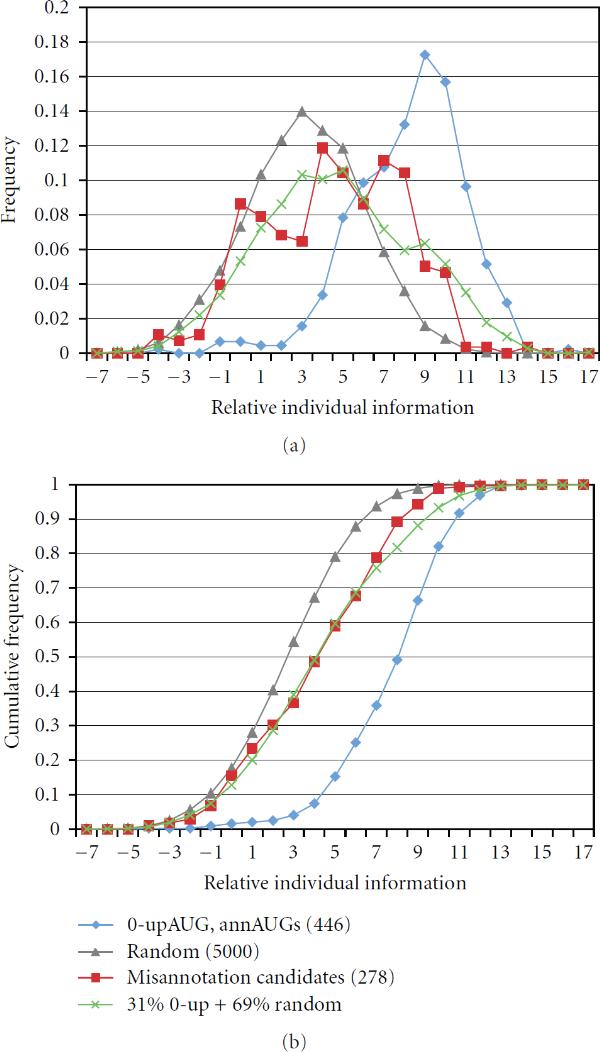
**Testing misannotation candidates**. TRII score distributions were examined for a set of 278 annAUGs that were likely to be misannotated based on sequence comparisons in 12 Drosophila species (red curve) [19–21]. Their score distribution (a) and cumulative distribution (b) are shifted toward the corresponding distributions for . The misannotation candidates distribution can be reconstructed by combining two distributions—0-upAUG and random—in proportions 31% and 69%, respectively, (green curve, see Methods).

**Figure 7:**
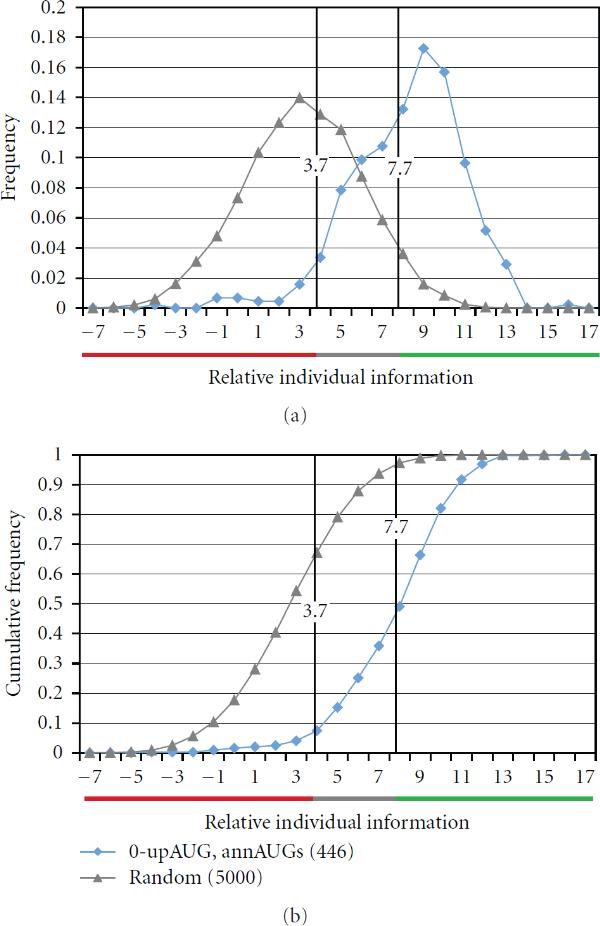
**Scoring thresholds**. The TRII score distribution (blue curve) for the high-confidence set of translation initiation sites  can be used as a reference curve for assessing translation start sites. Because 95% of the scores are higher than 3.7 bits, a score below this threshold can be considered nonconforming, and potentially weak or nonfunctional, with 95% confidence (red bar region). The score distribution (grey curve) for  shows 95% of scores below 7.7 bits. Scores above this threshold can be considered likely translation start sites with 95% confidence (green bar region). Scores between 3.7 and 7.7 could be functional or nonfunctional. In all cases, scores were calculated using the reference set .

In the examples in Figure 3, the reference set  and the test set  were chosen such that . Indeed, in choosing optimized reference sets, it is preferable if the reference and test sets are disjoint. As described in the Supplementary Material S.2.2, if , then test sequences in  have a slight scoring advantage compared to test sequences in the complement . Hence, in the analysis of translation-start relative individual information (TRII) score distributions described below (Figures 4–7) we tested sets of cDNAs with 5^'^UTR ≥ 200, using as a weight matrix reference set , the 1004 0-upAUG cDNAs with 5^'^UTRs between 100 and 199 in length. 

### 2.3. Validating Control Test Distributions

Using the improved weight matrices, we assessed the effectiveness of using score distributions of 0-upAUG sets as control test distributions for analysis of TRII scores. Comparisons of 0-upAUG distributions with distributions for sets of translation initiation sites from the Drosophila genome project support the use of 0-upAUG sets as representative of functional initiation sites. The Berkeley Drosophila Genome Project (BDGP) cDNA sequence set was constructed by sequencing high-quality, full-length cDNA libraries. The annotated ORFs and annAUGs were determined by finding the longest ORF encoded by each cDNA. The sequenced cDNAs (copies of mRNAs), which are part of the Drosophila Genome Project, can be compared with the set of annotated genes and their transcripts that has been assembled based initially on gene prediction algorithms. A subset of the cDNA ORFs that matched ORFs of annotated transcripts in the *Release 3* Drosophila genome were designated by BDGP as a "Gold collection" [11]. Gold collection ORFs were considered to be high-quality because they were both predicted in the genome and found in cDNAs. Comparison of the TRII score distributions for the full gold collection of cDNAs with 5^'^UTR ≥ 200 (red curve, Figure 4(a)) and the full set of *Release 5.9* predicted genes with 5^'^UTR ≥ 200 (green curve) reveals strikingly similar distributions. This is consistent with gold collection cDNAs being viewed as representative of current annotated gene models. The TRII score distributions for the Gold collection and *Release 5.9* predicted genes are both similar to the score distribution for the 0-upAUG set of cDNAs (blue curve), except that both have slightly greater frequencies of low-scoring start sites. We partitioned the Gold set cDNAs with 5^'^UTR ≥ 200 into two test subsets: those with no upAUGs, and those with 1 or more upAUGs. The 300 0-upAUG cDNAs in the Gold set have a distribution of TRII scores that is very similar to the distribution of the scores using  as a test set (red and blue curves, respectively, Figure 4(b)). These observations support the conclusion that the 0-upAUG annAUGs represent a high-confidence set of translation initiation sites and that various sets of 0-upAUG sites are appropriate to use for control test curves of TRII scores.

In this analysis, we noticed a disparity between TRII score distributions for experimentally observed cDNAs not in the Gold collection compared to Gold collection cDNAs that match predicted transcripts. TRII score distributions were compared using chi-square goodness of fit tests (Supplementary Material S.2.1). Various subsets of these "nongold" cDNAs (Figure 4) with at least one upAUG showed many more low-scoring annAUGs than their Gold counterparts, even though the nongold cDNAs appear to represent authentic mRNAs (see Figure 4 legend). The fact that nongold cDNAs represent mRNAs not in the predicted transcriptome suggests that the algorithms used to predict the Drosophila transcriptome prior to incorporation of cDNA data were conservative and failed to predict significant numbers of experimentally observed transcripts including mRNAs with upAUGs and low-scoring annAUGs.

### 2.4. Applications of Optimized TRII Scoring

We assessed the optimized TRII scoring method by analyzing the distributions of several special sets of interest in order to (1) assess upstream AUGs through comparisons with control distributions, and (2) assess nonconserved annAUGs using linear combinations of control curves.

#### 2.4.1. Upstream AUGs

As noted previously, many cDNAs have upAUGs in their 5^'^UTRs. We examined the TRII score distribution for the set of first AUGs upstream of the annAUG in gold collection cDNAs containing upAUGs (with 5^'^UTR ≥ 200). The distribution of TRII scores (green curve, Figure 5) was very similar to the random AUG set distribution (grey curve) suggesting that the upAUGs are generally weak or nonfunctional translation initiation sites. 

Nucleotide position −3 plays a central role in defining the consensus motif for translation initiation in Drosophila (see the final section on defining motifs). We observed that 57.6% of the upAUGs have C or U at this position, in contrast to only 7.6% of the annAUGs in the 0-upAUG set. Given that 47.5% of random sequences have C or U at this position (consistent with the background frequencies in 5^'^UTRs of 22.6% and 24.8% for C and U, resp.), this suggests that there may be some selection in favor of C or U at this position to reduce the likelihood of translation initiation at upAUGs. These observations suggest that the random sequence set is an appropriate comparison set to represent weak or nonfunctional AUGs in analysis of TRII score distributions. 

#### 2.4.2. Nonconserved annAUGs

The TRII score distributions for the 0-upAUG set of cDNAs and for the set of random sequences provide useful control test curves for assessing special sets of annAUGs. Linear combination of these control curves can be useful in cases where experimental distributions are intermediate between them. For example, we measured TRII scores for a set of annAUGs considered highly likely to be misannotated (red curve, Figure 6). These suspect annAUGs were marked for reannotation (Lin and Kellis, personal communication [19–21]) because their annAUG and downstream codons are not well conserved in 11 other Drosophila species that have been sequenced. The TRII score distribution for the suspect *Drosophila melanogaster* annAUGs was compared with the score distributions for  and . The relative individual information scores were calculated using the reference set .

As illustrated in Figure 6, the score distribution of the suspect set of annAUGs shows some similarity to the distribution for random sequences surrounding the AUG. This strongly supports the conclusion that many of the suspect annAUGs are either weak or nonfunctional translation initiation sites. 

In order to estimate the fraction of suspect annAUGs with random-like sequence context, we used a curve reconstruction approach. We compared the observed TRII score distribution of the suspect set (Figure 6, red curve) to a composite distribution (green curve) derived from the 0-upAUG (blue) and random (grey) curves combined in a ratio of 0.31 : 0.69. This ratio was chosen to minimize the sum of squares of differences between the corresponding values in the test (red) and composite (green) curves. Our analysis suggests that approximately 70% of the suspect annAUGs are misannotated or underannotated and about 30% are not misannotated. Therefore, while the majority of genes are correctly reannotated, some nonconserved annAUGs might be reannotated inappropriately based upon conservation assessment. This analysis illustrates the potential utility of reconstructing TRII score distributions as a linear combination of distributions for high-confidence (0-upAUG) and random sequences.

### 2.5. Estimating Confidence Intervals Using TRII Scores

The preceding analysis has established an optimized TRII scoring method and suggested that score distributions for 0-upAUG and random sequence sets provide valuable control test curves for assessing score distributions. In the next part of this study, we extended the interpretation of these control distributions. Because they can be used to represent high-confidence and weak or nonfunctional translation initiation sites, respectively, the control distributions can be treated as probability distributions to assess individual or groups of scores. Table 2 illustrates TRII scores corresponding to several probability thresholds for the score distributions of the random and 0-upAUG control test sets. If we consider the 0-upAUG set as representative of functional annAUGs, then we expect 95% of TRII scores to be above 3.7 bits, and only 5% to be below this threshold. Hence, an annAUG with a TRII score below 3.7 bits can be considered as weak or nonfunctional with 95% confidence. Comparison with the random sequence score distribution suggests that 95% of nonfunctional AUGs are expected to have scores below 7.7 bits. Hence, an AUG with a score above 7.7 bits can be considered as functional with 95% confidence. These two values define the confidence interval illustrated in Figure 7 (grey interval). The AUGs with scores between 3.7 and 7.7 bits may be either functional or nonfunctional. For example, for a TRII score threshold of 5.0, there are 85% of high-confidence start sites above this threshold (85% sensitivity), and 79% of random sequences are below this threshold (79% specificity; see Table 3 below). As discussed in Supplementary Material S.2.2, individual TRII scores can generally be considered reliable to within 0.6 to 0.8 bits.

**Table 2:** Score thresholds

	.05	.10	.50	.90	.95
TRIIthreshold_random_	−1.67	−0.56	3.19	6.82	7.75
TRIIthreshold_0upAUG_	3.71	4.89	8.40	10.74	11.27

*P* is the probability of obtaining the indicated TRII score or a lower score.

**Table 3:** Conditional probabilities for classification.

(a)	
**s**	**(start)**

	.00
−4	.00
−3	.00
−2	.00
−1	.01
0	.02
1	.02
2	.02
3	.04
4	.07
5	.15
6	.25
7	.36
8	.49
9	.66
10	.82
11	.92
12	.97
≥13	1.00

(b)	

s	(random)

≤−5	1.00
−4	.99
−3	.98
−2	.94
−1	.90
0	.82
1	.72
2	.60
3	.46
4	.33
5	.21
6	.12
7	.06
8	.03
9	.01
10	.00
11	.00
12	.00
≥13	.00

(start site ∣ TRII score ≤ s).

(random sequence ∣ TRII score ≥ s).

In our analysis above of annAUGs that were flagged as possibly misannotated due to poor conservation across species (Figure 6), 40% of the suspect annAUGs had scores below 3.7 bits, and only 19% of the suspect annAUGs have scores above 7.7 bits. The remaining 41% of the annAUGs had scores in the confidence interval between these thresholds.

The weight matrix used to calculate the TRII scores is provided in Supplementary Material S.3 and may be used to calculate scores for any AUG of interest. The TRII scores can also be calculated using a graphical user interface found at http://igs.wesleyan.edu/ > Databases and Tools > Information Theoretic Analysis (see Methods). The set of reference sequences  used to construct the weight matrix is provided in Supplementary Material S.1. The TRII scores for annAUGs of all predicted transcripts in the *Release 5.9 Drosophila melanogaster* genome are also provided in Supplementary Material S.1. 

In Table 3(a), we extend the analysis presented in Table 2 and Figure 7 to estimate the conditional probabilities, based on the distribution of TRII scores for , that a test sequence is a start site if it has a given TRII score or lower. Similarly, in Table 3(b), we estimate the conditional probabilities that a test sequence is random, and therefore weak or nonfunctional, if it has a given TRII score or higher. The latter conditional probabilities are based on the distribution of TRII scores for . Tables 3(a) and 3(b) provide a convenient summary for interpreting the TRII scores in Supplementary Material S.1.

The significant overlap in the TRII score distributions for random sequences and high-confidence initiation sites makes it necessary to treat intermediate TRII scores probabilistically as discussed above. Even though the distributions overlap, the TRII score measure can contribute to future algorithms for assessment of translation initiation in combination with other classifiers that incorporate properties such as RNA structure prediction [22] and sequence conservation [20].

The methods discussed to optimize TRII scoring—the utilization of high-confidence sets and probabilistic analysis of score distributions—can also be applied to the initiation context scoring method of Miyasaka [8]. The latter method has been used, for example, to predict and score translation initiation sites in a recent ribosome profiling study based on deep sequence analysis in yeast [9]. The Miyasaka method differs significantly from the TRII scoring approach since it uses a weight matrix of nucleotide frequency ratios computed relative to the frequency of the single most abundant nucleotide at each position. In contrast, each weight matrix entry for TRII scoring is the log of the nucleotide frequency at a position relative to the background frequency for that nucleotide (4). Both scoring methods give analogous score distributions for  and  allowing probabilistic assessment of scores (data not shown). However, the TRII scoring method has the advantage that it measures more transparently the deviations from background nucleotide frequencies that have been selected during evolution of functional sites.

### 2.6. Defining Motifs Using a Consensus Matrix

**Figure 8:**
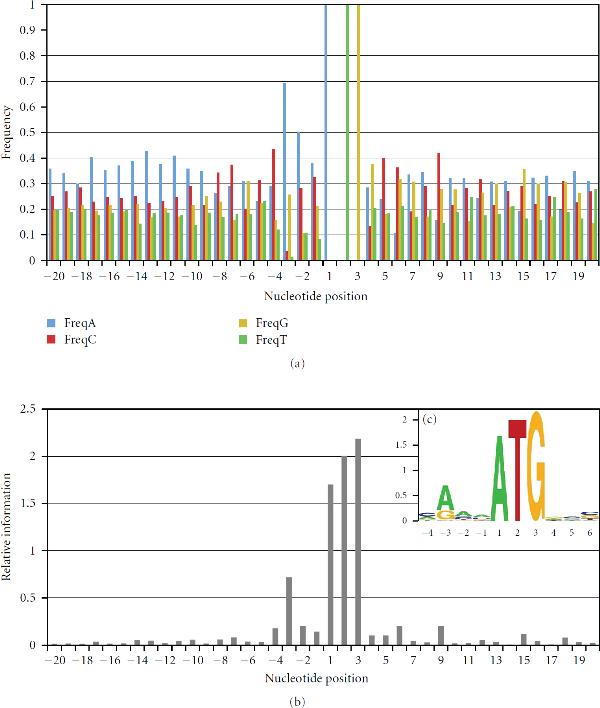
**Nucleotide frequencies and relative information**. (a) Nucleotide frequencies are graphed for  excluding 22 (5%) of these sequences with relative individual information scores below 3.71 bits. (b) Relative information graph for the same set of cDNAs. Note the relative information at nucleotide position −3 where C and U are depressed, and A is elevated. (c) The positional logo for positions −4 to 6 is illustrated. Figure 9 shows the corresponding weight matrix.

In addition to optimizing the TRII scoring method, the 0-upAUG high-confidence sets were used to improve assessment of nucleotide preferences at translation initiation sites. In particular, the optimized high-confidence sets of annotated translation start sites were used to assess sequence conservation at initiation sites and to compare this conservation with previous descriptions of consensus sequences [23, 24]. Figure 8 shows the nucleotide frequencies and corresponding relative information profiles for an optimized 0-upAUG set consisting of  from which the 22 sequences (5%) with lowest TRII scores have been excluded to remove outliers. These excluded sequences contain some start sites with negative individual information scores that are postulated to be nonfunctional based on thermodynamic considerations [25]. The relative information profile (Figure 8(b)) shows that in addition to the high relative information (relative entropy) at the AUG, there is also significant relative information at positions −4 to −1, in particular at −3. There is also elevated relative information at positions 4 and 5 (positions downstream of 5 are discussed later).

**Figure 9:**
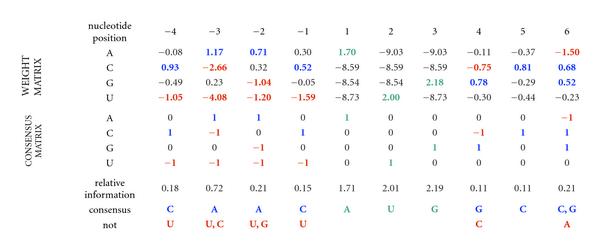
**Weight and consensus matrices**. Weights show values used to calculate relative individual scores. Each weight was calculated using the expression  where  is the observed frequency,  is the background frequency, and  is the sampling correction. To calculate TRII scores, the weights corresponding to the nucleotide present at each position in a sequence are summed. The observed frequencies are derived from , excluding 22 (5%) of these sequences with relative individual information scores below 3.71 bits. The background frequencies are calculated from the 5′UTRs of 8,607 cDNAs. Color Coding: Blue (weight ≥ 0.5), Red (weight ≤ −0.5), Green (fixed AUG).

This optimized 0-upAUG set (Figure 8) was used to create a weight matrix consisting of the values [, or ,; compare with (4)] that illustrates which nucleotide choices are particularly important in the translational initiation sites (Figure 9). The weights ≥0.5 are indicated in blue and the weights ≤−0.5 are indicated in red. These thresholds can be used to compute a consensus matrix as illustrated in Figure 9. The nucleotide choices with weights ≥0.5 define the following consensus sequence for translation initiation: (5)

where  denotes " or ". This consensus is similar to that described earlier for Drosophila translation start sites [26, 27]. However, Cavener describes A as the consensus nucleotide for position −1. While A is slightly more abundant at this position (Figure 8(a)), when compared to the background frequencies of 5^'^UTRs, the elevation in C at this position is more pronounced (Figure 9). This suggests that a ribosome scanning a 5^'^UTR favors a C at this position. 

The preceding approach for defining a consensus sequence does not take into account the importance of the *absence* of nucleotides at certain positions—those nucleotide choices that receive a weight ≤−0.5 (red in Figure 9). For example, U should be avoided at any position −4 to −1. The disruptive effect on translation initiation of having U at position −3 has been noted before [28, 29]. Hence, as summarized in Figure 9, a more useful description of the consensus would be(6)

where  denotes " and not  and not ". Using this approach, a weight  indicates that  and a weight ≤−0.5 indicates that . Hence, the "consensus" that is defined represents nucleotides whose frequencies are at least 1.41 fold higher than their background frequency. Similarly, the "not N" consensus choices have frequencies that are at least 1.41 fold lower than background. Defining the consensus measure based on deviations from background frequencies provides a natural indication of the nucleotide preferences of the translation machinery. Indeed, the most pronounced deviations are for  and  at position −3 (6.5 and 17.7 fold lower than background, resp.), indicating that the presence of either of these pyrimidine nucleotides at this position is particularly deleterious, and that their exclusion is one of the key hallmarks of a functional translation initiation site. 

Examining the region downstream of nucleotide position 5 reveals that relative information values are elevated at positions 6, 9, 15, and 18. As discussed previously [30, 31], a 3-base periodicity is characteristic of open reading frames. Relative information is elevated at each of these positions, because  is depressed, and  and  are elevated (see Figure 9 position 6, Figure 8, and Supplementary Tables 3 and 4). The periodic elevation of relative information and the corresponding weights indicate that these positions positively contribute to the translation-start relative individual information (TRII) scores. Indeed, if TRII scores are calculated using positions −20 to 40 (data not shown), the distribution of scores is shifted to the right, and the scoring is better able to distinguish between the 0-upAUG control test set and sets of putative nonfunctional start sites (e.g., the set in Figure 6 discussed above). Statistical analysis of weight matrices is described in Supplementary Material S.3 and Supplementary Table 2. 

Note that each expression  represents the  of the probability that a given nucleotide  will occur relative to its background probability, and the summing of these log terms represents the product of these probabilities which is the overall probability of a given individual sequence (the TRII score without a sampling correction). Hence, the weight matrix captures the essence of the consensus notion from a probability perspective.

Using a weight matrix to represent a consensus sequence is a natural extension of Schneider and colleagues' use of the weight matrix for sequence walkers [32–34]. The positional weight matrix (Figure 9) provides a fuller view of the consensus than the sequence logo format (Figure 8(c)) which is commonly used to represent a sequence consensus. Unlike a sequence logo, the positional weight matrix explicitly conveys deviations from background frequencies showing when nucleotides are underrepresented (negative matrix entries) or overrepresented (positive entries). 

## 3. Conclusions

A TRII scoring method based on high-confidence translation initiation sites has been developed to assess translation initiation sites. The 0-upAUG high-confidence sets are used to compute the TRII scoring weight matrix as well as to provide control test curves which, in addition to random sequence score distributions, allow for probabilistic assessment of individual TRII scores. In addition, comparison with control test curves gives powerful methods to analyze TRII score distributions for groups of translation initiation sites of special interest. The 0-upAUG high-confidence sets also provide improved quantitative descriptions of the consensus motif for translation initiation in Drosophila. TRII score analysis of cDNAs containing upAUGs suggests that further experimental analysis of this class of cDNAs is warranted to assess their annotated translation initiation sites.

## 4. Methods

### 4.1. Translation Relative Individual Information (TRII) Scoring

The collections of genomic and cDNA sequences were stored in a relational database. The database schema is illustrated in Supplementary Figure 4. Information-theoretic calculations were performed using a variety of stored procedures in the database. A listing of the control test set of 0-upAUG start sites at positions −20 to 20 in sequences with 5^'^UTRs ≥ 200, and their relative individual information (TRII) scores, are provided in Supplementary Material S.1.2. These TRII scores are based on using the reference set . 

As described in the Introduction, relative individual information was calculated using the expression (7)

where the sampling correction  was estimated as described previously [3, 4] assuming background frequencies of 0.25 for each nucleotide. In particular, we used the theoretical estimate of  for . If the actual 5^'^UTR background frequencies are used to estimate , the value increases by less than 0.00003 for .

### 4.2. Reconstruction of TRII Score Distributions

We estimated the fraction  of AUG sites in a test set that were similar to optimized translation initiation sites and therefore likely to be functional (see, e.g., Figure 6) as follows: given , construct a new distribution using the values , where  and  denote two TRII score distributions, and  represents an individual score (of a bin). Then choose the fraction  that *minimizes* the sum of the differences squared between these values and the values of the actual test set distribution . For our computations, the distribution  was based on the scores for  and  was based on the scores for  (Table 1) or  (Figure 7). 

### 4.3. Information Calculator

We provide a web interface for performing calculations on sets of inputed aligned sequences (http://igs.wesleyan.edu/ > Databases and Tools). The interface generates a weight matrix from the aligned sequences so that relative information values and relative individual information scores can be calculated for sequences of interest. The interface can be used to assess potential translation initiation sites, or other kinds of motifs for which sets of aligned sequences with the motif are available. 

## List of Abbreviations

TRII: Translation relative individual information; ORF: Open reading frame; BDGP: Berkeley drosophila genome project; upAUG: Upstream AUG; annAUG: Annotated AUG; UTR: Untranslated region.

## Supplementary Material

## Supplementary Material

Supplementary Material 1 The Supplemental Materials include descriptions of statistical tests to compare TRII score distributions, individual TRII scores, and weight matrices. Weight matrices computed with various reference sets are provided, as well as a schema for the database used in this study, and a summary of the gene sets used for analysis. The Supplemental Materials close with results from a parallel analysis performed in *Saccharomyces cerevisiae*. An additional supplemental file provides the set of reference sequences *S*_100-199_ used to construct the weight matrix, and score distributions for the 0-upAUG control test set *S*_200_ and the random test sets *S*_rand_. Another supplemental file provides TRII scores for annAUGs of all predicted transcripts in the *Release 5.9 Drosophila melanogaster* genome. Click here for file

Supplementary Material 2 Click here for file

Supplementary Material 3 Click here for file
