# Supplementary material for: TRII: A Probabilistic Scoring of Drosophila melanogaster Translation Initiation Sites
Source: EURASIP J Bioinform Syst Biol. 2010 Oct 18;2010(1):814127. doi: 10.1155/2010/814127 (PMC3171364; doi:10.1155/2010/814127)
Supplement: Supplementary Material 1 — The Supplemental Materials include descriptions of statistical tests to compare TRII score distributions, individual TRII scores, and weight matrices. Weight matrices computed with various reference sets are provided, as well as a schema for the database used in this study, and a summary of the gene sets used for analysis. The Supplemental Materials close with results from a parallel analysis performed in Saccharomyces cerevisiae. An additional supplemental file provides the set of reference sequences S100-199 used to construct the weight matrix, and score distributions for the 0-upAUG control test set S200 and the random test sets Srand. Another supplemental file provides TRII scores for annAUGs of all predicted transcripts in the Release 5.9 Drosophila melanogaster genome. [file 1687-4153-2010-814127-S1.pdf]

**TRII: A Probabilistic Scoring of *Drosophila melanogaster* Translation Initiation Sites**

Michael P. Weir [1] and Michael D. Rice [2]

Department of Biology [1] and
Department of Mathematics and Computer Science [2]
Wesleyan University
Middletown, CT 06459
USA

## SUPPLEMENTAL MATERIALS

### S.1 Supplemental files

### S.1.1 TRII scores of Release 5.9 transcriptome

Supplementary file Rel5_9TranscriptomeTRII_Scores.xls contains TRII scores for annotated AUGs of all transcripts in the Release 5.9 *Drosophila melanogaster* genome. The reference set $S_{100\text{-}199}$ consisting of 1004 0-upAUG cDNA sequences with 5'UTR lengths between 100 and 199 is also listed.

### S.1.2 Weight matrix, control test curve sequences, and TRII scores

Supplementary file S1.2_supplMat_sequencesets.xls contains the sequences for the control test set $S_{200}$ and the distributions of scores for $S_{200}$ and $S_{rand}$.

## S.2 Statistical evaluation of TRII scores

## S.2.1 Comparison of TRII score distributions

Two distributions of TRII scores can be compared to assess whether they can be considered to be from the same distribution. A chi-square goodness of fit test can be used for this comparison of distributions. Supplemental Table 1 illustrates an example using data from Fig. 4B.

**Supplemental Table 1**

| OBSERVED | TRII score bin | ≥1upAUG, BDGP Gold | ≥1upAUG, not BDGP Gold | Sum |
|---|---|---|---|---|
| | ≤ -1 | 18 | 51 | 69 |
| | 0 | 15 | 47 | 62 |
| | 1 | 29 | 72 | 101 |
| | 2 | 38 | 97 | 135 |
| | 3 | 57 | 120 | 177 |
| | 4 | 67 | 153 | 220 |
| | 5 | 101 | 153 | 254 |
| | 6 | 137 | 182 | 319 |
| | 7 | 159 | 201 | 360 |
| | 8 | 175 | 166 | 341 |
| | 9 | 185 | 166 | 351 |
| | 10 | 147 | 125 | 272 |
| | 11 | 125 | 74 | 199 |
| | 12 | 64 | 43 | 107 |
| | ≥ 13 | 32 | 25 | 57 |
| | | 1349 | 1675 | 3024 |

| EXPECTED | | | |
|---|---|---|---|
| | ≤ -1 | 30.78 | 38.22 |
| | 0 | 27.66 | 34.34 |
| | 1 | 45.06 | 55.94 |
| | 2 | 60.22 | 74.78 |
| | 3 | 78.96 | 98.04 |
| | 4 | 98.14 | 121.86 |
| | 5 | 113.31 | 140.69 |
| | 6 | 142.31 | 176.69 |
| | 7 | 160.60 | 199.40 |
| | 8 | 152.12 | 188.88 |
| | 9 | 156.58 | 194.42 |
| | 10 | 121.34 | 150.66 |
| | 11 | 88.77 | 110.23 |
| | 12 | 47.73 | 59.27 |
| | ≥ 13 | 25.43 | 31.57 |

| Chi-square calculations | | | $\sum \frac{(obs\text{-}exp)^2}{exp}$ |
|---|---|---|---|
| ≤ -1 | 5.31 | 4.27 | |
| 0 | 5.79 | 4.67 | |
| 1 | 5.72 | 4.61 | |
| 2 | 8.20 | 6.60 | |
| 3 | 6.11 | 4.92 | |
| 4 | 9.88 | 7.96 | |
| 5 | 1.34 | 1.08 | |
| 6 | 0.20 | 0.16 | |
| 7 | 0.02 | 0.01 | |
| 8 | 3.44 | 2.77 | |
| 9 | 5.16 | 4.15 | |
| 10 | 5.43 | 4.37 | |
| 11 | 14.78 | 11.91 | |
| 12 | 5.54 | 4.47 | |
| ≥ 13 | 1.70 | 1.37 | |
| | | **chi-sq =** | **141.93** |

The TRII scores are distributed into 15 bins (d.f. = 14), consolidating counts in trailing parts of the curves into bins with greater than 10 counts. The expected counts are computed based on the sums of bin counts in the two distributions. For 14 degrees of freedom, the chi-square statistic

$$\sum (obs\text{-}exp)^2/exp$$

is expected to be ≤ 29.14 ($p$ = 0.99). Since the chi-square test statistic in the above example is 141.93, this suggests that the distributions for the Gold and non-Gold cDNA sets with ≥ 1 upAUG (Fig. 4B) are not equivalent by this test.

## S.2.2 Analysis of individual scores

An idealized implementation of TRII scoring would use a large reference set for weight matrix construction that is highly representative of an optimal functional site. Although growing genomic datasets are beginning to approach this goal, in practice, the inevitable suboptimality of reference sets necessitates statistical evaluations of TRII scores.

To investigate variability of individual TRII scores, we examined TRII scores using multiple reference sets $R$ drawn from the same source set. Each of the 446 control test sequences in $S_{200}$ was scored using 100 different reference sets $R$ of size $m$ selected randomly without replacement from $S_{100\text{-}199}$. We examined the distribution of scores for each test sequence. For example, for $m$ = 500, the mean standard deviation for the 446 distributions was 0.335 bits (Suppl. Fig 1A); the distribution of standard deviations is shown in Suppl Fig. 1B (blue curve). In addition, 95% of the scores were within 0.64 ± 0.09 bits of the mean score for each sequence; examples of the range of scores for several sequences are illustrated in Suppl. Fig. 1C. Hence, when considering individual scores with reference sets of size 500, this analysis suggests that scores are generally fairly consistent but occasionally vary by as much as 0.6 to 0.8 bits from the mean. A similar analysis for different

values of $m$ indicates that smaller standard deviations are associated with larger reference sets (Suppl. Fig. 1A). For example, less variation in TRII scores was observed for $m$ = 750 (Suppl. Fig 1A, B, D). We note that for our analyses, we used the large reference set $S_{100\text{-}199}$ of size 1004.

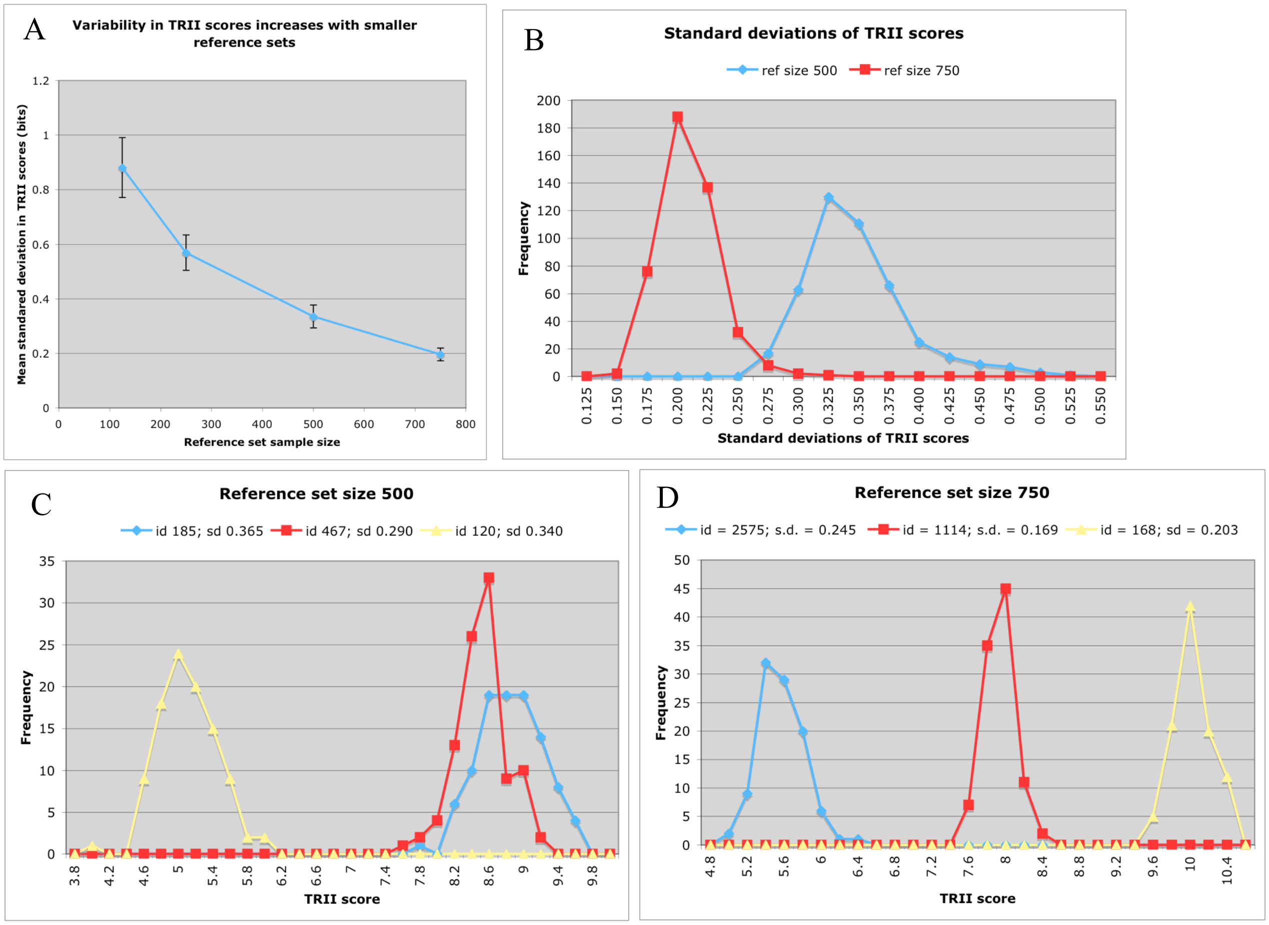


**Supplemental Fig. 1** Assessing variability of TRII scores (see text).

When groups of sequences are considered, rather than individual sequences, the TRII score distributions are quite reliable. For example, when disjoint test $T$ and reference $R_{nonoverlap}$ sets of size 500 are randomly chosen without replacement from 1,450 0-upAUG source sites with □UTR $\geq$ 100, the mean TRII score of the test sets for 50 trials is 7.90 $\pm$ 0.12 bits (Suppl. Fig. 2A).

However, this analysis reveals that choosing test sequences from the reference set leads to higher mean scores. For example, when test sets of size 500 are scored using a reference set equal to the test set ($T = R_{overlap}$), the mean TRII score is 8.22 $\pm$ 0.10 bits. However, the disparity in scores using $R_{nonoverlap}$ and $R_{overlap}$ is lower for larger reference sets (Suppl. Fig. 2A). For example, if the test set has size 400 and the reference set has size 1000, the average TRII scores differ by only 0.16 bits (8.05 $\pm$ 0.11 bits when $T \cap R_{nonoverlap} = \varnothing$ compared to 8.21 $\pm$ 0.10 bits when $T \subset R_{overlap}$ (Suppl. Fig. 2B). Similar analysis was performed using reference and test sets of 500 with varying degrees of overlap (Suppl. Fig. 3).

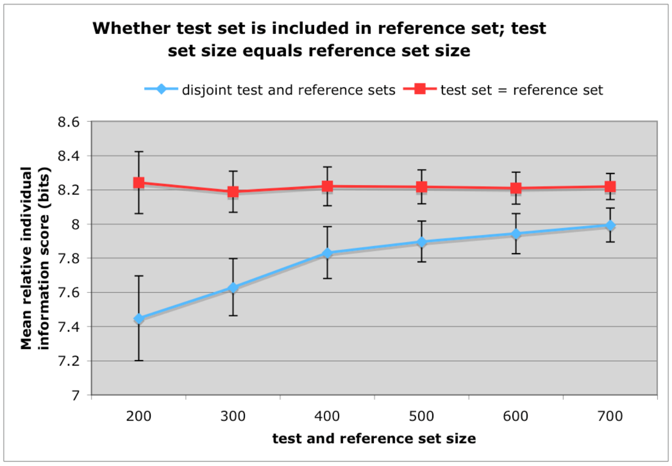


A

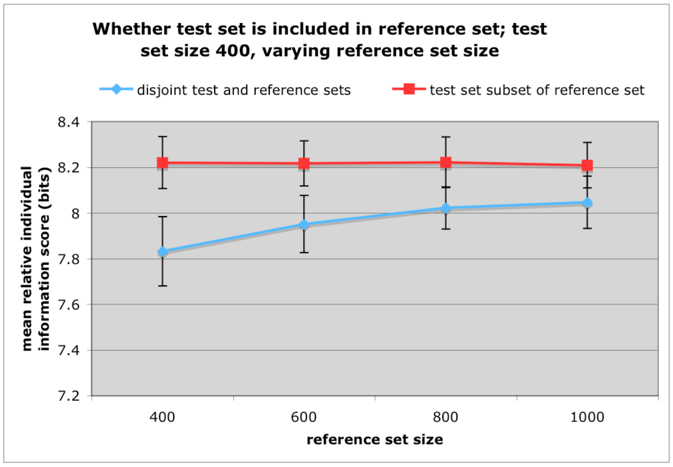


B

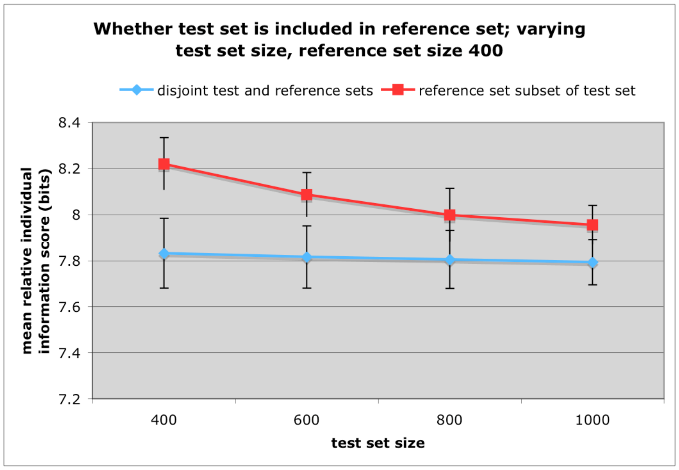


C

**Supplemental Fig. 2.** In each of 50 independent trials, a test set $T$ and two reference sets $R_{overlap}$ and $R_{nonoverlap}$ were chosen randomly from a set of 1450 annAUG sequences (positions -20 to 20 in 0-upAUGs with $5'$UTR $\geq$ 100) such that (i) $T \subseteq R_{overlap}$ and (ii) $T \cap R_{nonoverlap} = \emptyset$. The mean TRII scores for the test set $T$ were calculated using both $R_{nonoverlap}$ (blue) and $R_{overlap}$ (red). The means of the 50 trial means are graphed. Mean TRII scores of set $T$ are higher in case (i) because the test sequences themselves contribute to the weight matrix. TRII scores for set $T$ are lower in case (ii); this effect is more pronounced for smaller sizes of reference sets. Error bars indicate standard deviations of the means.

A. Reference and test sets of equal size

B. Constant test set size; varying reference set size

C. Varying test set size, constant reference set size

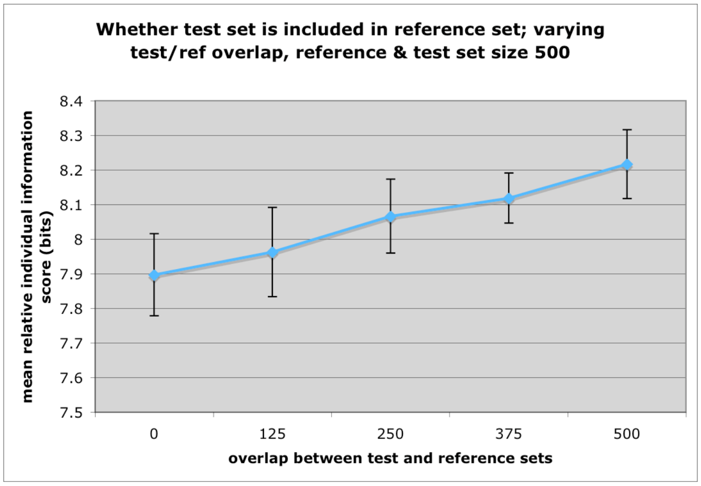


**Supplemental Fig. 3.** In an analysis similar to the one used for Supplemental Fig. 2, we tested reference and test sets of 500 sequences with varying amounts of overlap between 0 ($T \cap R_{nonoverlap} = \varnothing$) and 500 ($T = R$).

## S.3 Weight Matrix Analysis

We examined how multiple random sampling of reference sequences affects weight matrices.

We chose 100 random samples from $S_{100\text{-}199}$ each consisting of 500 reference sequences. For each of these reference sets, we constructed a weight matrix and calculated the score for each of the 446 test sequences in $S_{200}$. The standard deviations were calculated for the score distributions for each of the 446 sequences, and the mean and standard deviation of this distribution of standard deviations is reported in Supplemental Table 2.

Supplemental Table 3 shows the weight matrix used to calculate TRII scores in Figs. 4, 6-8. It is constructed from the reference set $S_{100\text{-}199}$.

Supplemental Table 4 shows the consensus matrix illustrated in Table 4 of the paper, based on the set of 446 sequences in $S_{200}$ but excluding 22 sequences with TRII scores below 3.71 (see Fig. 7).

Supplemental Table 5 shows a weight matrix based on the set of 3,470 sequences in $U_{200}$, the set of cDNAs with 5'UTR ≥ 200.

**Supplemental Table 2** (based on samples from $S_{100\text{-}199}$)

| nt position | -20 | -19 | -18 | -17 | -16 | -15 | |
|---|---|---|---|---|---|---|---|
| A | 0.18 | 0.12 | 0.18 | 0.29 | 0.20 | 0.22 | WEIGHTS |
| C | -0.13 | 0.03 | 0.20 | 0.03 | 0.00 | 0.06 | |
| G | -0.09 | -0.12 | -0.34 | -0.33 | -0.24 | -0.35 | |
| T | -0.08 | -0.12 | -0.21 | -0.21 | -0.11 | -0.11 | |
| A | 0.07 | 0.06 | 0.07 | 0.06 | 0.06 | 0.06 | SDEV |
| C | 0.09 | 0.08 | 0.08 | 0.08 | 0.09 | 0.08 | |
| G | 0.09 | 0.10 | 0.10 | 0.10 | 0.10 | 0.09 | |
| T | 0.09 | 0.08 | 0.09 | 0.09 | 0.09 | 0.09 | |

| nt position | -14 | -13 | -12 | -11 | -10 | -9 | -8 | -7 | -6 | -5 | |
|---|---|---|---|---|---|---|---|---|---|---|---|
| A | 0.28 | 0.38 | 0.30 | 0.39 | 0.22 | 0.13 | -0.07 | -0.03 | -0.02 | -0.32 | WEIGHTS |
| C | -0.04 | -0.02 | -0.02 | 0.02 | 0.16 | 0.15 | **0.51** | 0.38 | -0.15 | 0.46 | |
| G | -0.14 | -0.43 | -0.27 | -0.27 | -0.26 | -0.11 | -0.31 | -0.28 | 0.31 | -0.10 | |
| T | -0.28 | -0.22 | -0.22 | -0.44 | -0.28 | -0.27 | -0.28 | -0.17 | -0.19 | -0.09 | |
| A | 0.06 | 0.06 | 0.06 | 0.05 | 0.07 | 0.06 | 0.07 | 0.07 | 0.07 | 0.08 | SDEV |
| C | 0.08 | 0.08 | 0.09 | 0.09 | 0.09 | 0.08 | **0.06** | 0.07 | 0.09 | 0.07 | |
| G | 0.10 | 0.11 | 0.10 | 0.10 | 0.10 | 0.09 | 0.10 | 0.09 | 0.09 | 0.09 | |
| T | 0.09 | 0.08 | 0.09 | 0.09 | 0.09 | 0.09 | 0.10 | 0.09 | 0.09 | 0.07 | |

| nt position | -4 | -3 | -2 | -1 | 1 | 2 | 3 | 4 | 5 | 6 | |
|---|---|---|---|---|---|---|---|---|---|---|---|
| A | 0.07 | **1.09** | **0.72** | 0.42 | **1.70** | -9.27 | -9.27 | -0.26 | -0.21 | **-0.85** | WEIGHTS |
| C | **0.91** | **-2.07** | 0.27 | 0.36 | -8.83 | -8.83 | -8.83 | **-0.62** | **0.82** | 0.39 | |
| G | **-0.67** | 0.19 | **-1.10** | -0.04 | -8.78 | -8.78 | **2.19** | **0.72** | -0.44 | 0.49 | |
| T | **-1.17** | **-2.69** | **-1.09** | **-1.60** | -8.96 | **2.00** | -8.96 | -0.09 | **-0.54** | -0.17 | |
| | | | | | | | | | | | |
| A | 0.07 | **0.03** | **0.05** | 0.05 | 0.00 | 0.00 | 0.00 | 0.07 | 0.07 | **0.10** | SDEV |
| C | **0.06** | **0.18** | 0.08 | 0.08 | 0.00 | 0.00 | 0.00 | **0.11** | **0.06** | 0.07 | |
| G | **0.12** | 0.08 | **0.13** | 0.09 | 0.00 | 0.00 | 0.00 | **0.05** | 0.10 | 0.07 | |
| T | **0.14** | **0.25** | **0.13** | **0.15** | 0.00 | 0.00 | 0.00 | 0.07 | **0.10** | 0.08 | |

| nt position | 7 | 8 | 9 | 10 | 11 | 12 | 13 | 14 | 15 | 16 | |
|---|---|---|---|---|---|---|---|---|---|---|---|
| A | 0.03 | 0.12 | **-0.80** | 0.06 | 0.02 | -0.47 | -0.11 | 0.02 | **-0.73** | 0.03 | WEIGHTS |
| C | -0.07 | 0.04 | **0.81** | -0.01 | 0.11 | 0.42 | 0.19 | 0.03 | 0.25 | -0.12 | |
| G | 0.36 | -0.05 | 0.25 | 0.32 | -0.43 | 0.27 | 0.37 | -0.11 | **0.62** | 0.48 | |
| T | -0.43 | -0.19 | **-0.63** | -0.48 | 0.16 | -0.27 | **-0.54** | 0.02 | -0.28 | **-0.56** | |
| | | | | | | | | | | | |
| A | 0.07 | 0.06 | **0.10** | 0.07 | 0.07 | 0.10 | 0.07 | 0.07 | **0.11** | 0.06 | SDEV |
| C | 0.09 | 0.09 | **0.05** | 0.08 | 0.08 | 0.07 | 0.08 | 0.08 | 0.08 | 0.09 | |
| G | 0.08 | 0.10 | 0.08 | 0.07 | 0.10 | 0.08 | 0.08 | 0.09 | **0.06** | 0.06 | |
| T | 0.10 | 0.08 | **0.11** | 0.09 | 0.07 | 0.10 | **0.11** | 0.08 | 0.09 | 0.10 | |

| nt position | 17 | 18 | 19 | 20 | |
|---|---|---|---|---|---|
| A | 0.07 | **-0.60** | 0.05 | -0.05 | WEIGHTS |
| C | 0.02 | 0.38 | 0.03 | 0.03 | |
| G | -0.35 | **0.53** | 0.46 | -0.47 | |
| T | 0.13 | -0.43 | **-0.75** | 0.32 | |
| | | | | | |
| A | 0.06 | **0.08** | 0.07 | 0.06 | SDEV |
| C | 0.07 | 0.07 | 0.09 | 0.09 | |
| G | 0.10 | **0.07** | 0.08 | 0.11 | |
| T | 0.07 | 0.11 | **0.11** | 0.07 | |

**Supplemental Table 3** (based on the full set $S_{100\text{-}199}$)

| nt position | -20 | -19 | -18 | -17 | -16 | -15 |
|---|---|---|---|---|---|---|
| A | 0.18 | 0.14 | 0.20 | 0.30 | 0.20 | 0.23 |
| C | -0.11 | 0.03 | 0.21 | 0.03 | 0.01 | 0.06 |
| G | -0.09 | -0.11 | -0.34 | -0.32 | -0.23 | -0.35 |
| U | -0.08 | -0.13 | -0.22 | -0.21 | -0.10 | -0.11 |

| nt position | -14 | -13 | -12 | -11 | -10 | -9 | -8 | -7 | -6 | -5 |
|---|---|---|---|---|---|---|---|---|---|---|
| A | 0.28 | 0.38 | 0.30 | 0.39 | 0.22 | 0.13 | -0.06 | -0.01 | -0.01 | -0.31 |
| C | -0.03 | -0.03 | -0.02 | 0.03 | 0.17 | 0.15 | **0.51** | 0.39 | -0.15 | 0.47 |
| G | -0.13 | -0.41 | -0.25 | -0.27 | -0.26 | -0.10 | -0.28 | -0.29 | 0.32 | -0.09 |
| U | -0.28 | -0.22 | -0.22 | -0.44 | -0.28 | -0.27 | -0.30 | -0.18 | -0.19 | -0.10 |

| nt position | -4 | -3 | -2 | -1 | 1 | 2 | 3 | 4 | 5 | 6 |
|---|---|---|---|---|---|---|---|---|---|---|
| A | 0.08 | **1.10** | **0.72** | 0.43 | **1.70** | -9.98 | -9.98 | -0.26 | -0.21 | **-0.84** |
| C | **0.91** | **-2.04** | 0.27 | 0.36 | -9.98 | -9.98 | -9.98 | **-0.62** | **0.83** | 0.39 |
| G | **-0.65** | 0.18 | **-1.09** | -0.03 | -9.98 | -9.98 | **2.19** | 0.72 | -0.45 | **0.50** |
| U | **-1.17** | **-2.67** | **-1.07** | **-1.60** | -9.98 | **2.01** | -9.98 | -0.08 | **-0.54** | -0.18 |

| nt position | 7 | 8 | 9 | 10 | 11 | 12 | 13 | 14 | 15 | 16 |
|---|---|---|---|---|---|---|---|---|---|---|
| A | 0.05 | 0.12 | **-0.78** | 0.06 | 0.01 | -0.46 | -0.10 | 0.01 | **-0.75** | 0.05 |
| C | -0.06 | 0.04 | **0.82** | -0.01 | 0.12 | 0.43 | 0.19 | 0.05 | 0.26 | -0.11 |
| G | 0.36 | -0.03 | 0.25 | 0.32 | -0.42 | 0.27 | 0.37 | -0.11 | **0.63** | 0.47 |
| U | -0.45 | -0.18 | **-0.63** | -0.46 | 0.17 | -0.26 | **-0.55** | 0.02 | -0.27 | **-0.55** |

| nt position | 17 | 18 | 19 | 20 |
|---|---|---|---|---|
| A | 0.07 | **-0.59** | 0.05 | -0.04 |
| C | 0.03 | 0.38 | 0.03 | 0.03 |
| G | -0.36 | 0.53 | 0.46 | -0.44 |
| U | 0.14 | -0.42 | **-0.74** | 0.32 |

**Supplemental Table 4** (based on the subset of $S_{200}$ with the top 95% of TRII scores)

| nt position | -20 | -19 | -18 | -17 | -16 | -15 |
|---|---|---|---|---|---|---|
| A | 0.22 | 0.14 | -0.04 | 0.39 | 0.20 | 0.27 |
| C | 0.14 | 0.24 | 0.33 | 0.01 | 0.11 | 0.10 |
| G | -0.17 | -0.11 | -0.03 | -0.18 | -0.03 | -0.20 |
| U | -0.35 | -0.40 | -0.31 | **-0.51** | -0.42 | -0.35 |

| nt position | -14 | -13 | -12 | -11 | -10 | -9 | -8 | -7 | -6 | -5 |
|---|---|---|---|---|---|---|---|---|---|---|
| A | 0.33 | 0.46 | 0.29 | 0.41 | 0.22 | 0.18 | -0.23 | -0.10 | 0.02 | -0.41 |
| C | 0.15 | -0.02 | 0.02 | 0.12 | 0.35 | -0.08 | **0.59** | **0.71** | -0.18 | 0.46 |
| G | 0.00 | -0.39 | -0.10 | -0.37 | -0.03 | 0.19 | 0.06 | -0.47 | 0.49 | 0.03 |
| U | **-0.81** | -0.44 | -0.42 | **-0.51** | **-0.86** | -0.42 | **-0.57** | -0.46 | -0.47 | -0.11 |

| nt position | -4 | -3 | -2 | -1 | 1 | 2 | 3 | 4 | 5 | 6 |
|---|---|---|---|---|---|---|---|---|---|---|
| A | -0.08 | **1.17** | **0.71** | 0.30 | **1.70** | -8.74 | -8.74 | -0.11 | -0.37 | **-1.50** |
| C | **0.93** | **-2.66** | 0.32 | **0.52** | -8.74 | -8.74 | -8.74 | **-0.75** | **0.81** | **0.68** |
| G | -0.50 | 0.23 | **-1.05** | -0.05 | -8.74 | -8.74 | **2.18** | **0.78** | -0.29 | **0.52** |
| U | **-1.05** | **-4.08** | **-1.20** | **-1.59** | -8.74 | **2.00** | -8.74 | -0.30 | -0.44 | -0.23 |

| nt position | 7 | 8 | 9 | 10 | 11 | 12 | 13 | 14 | 15 | 16 |
|---|---|---|---|---|---|---|---|---|---|---|
| A | 0.12 | 0.16 | **-0.98** | 0.06 | 0.06 | -0.34 | 0.00 | 0.01 | **-0.67** | 0.07 |
| C | -0.25 | 0.35 | **0.88** | -0.08 | 0.30 | 0.49 | -0.08 | 0.25 | 0.34 | -0.05 |
| G | 0.48 | -0.37 | 0.34 | 0.33 | **-0.52** | 0.27 | 0.45 | -0.08 | **0.70** | 0.45 |
| U | **-0.57** | -0.35 | **-0.77** | -0.40 | -0.02 | **-0.51** | -0.47 | -0.23 | **-0.61** | **-0.66** |

| nt position | 17 | 18 | 19 | 20 |
|---|---|---|---|---|
| A | 0.10 | **-0.63** | 0.18 | 0.01 |
| C | 0.15 | 0.44 | -0.01 | 0.24 |
| G | -0.37 | 0.47 | 0.25 | **-0.61** |
| U | -0.01 | -0.40 | **-0.61** | 0.16 |

**Supplemental Table 5** (based on the full set $U_{200}$)

| nt position | -20 | -19 | -18 | -17 | -16 | -15 |
|---|---|---|---|---|---|---|
| A | 0.11 | -0.02 | 0.06 | 0.18 | 0.04 | 0.14 |
| C | 0.10 | 0.26 | 0.12 | 0.10 | 0.24 | 0.06 |
| G | -0.03 | 0.01 | 0.07 | -0.13 | -0.07 | 0.04 |
| U | -0.22 | -0.27 | -0.29 | -0.26 | -0.25 | -0.31 |

| nt position | -14 | -13 | -12 | -11 | -10 | -9 | -8 | -7 | -6 | -5 |
|---|---|---|---|---|---|---|---|---|---|---|
| A | 0.18 | 0.08 | 0.17 | 0.33 | 0.04 | -0.02 | -0.08 | -0.14 | -0.05 | -0.13 |
| C | 0.02 | 0.19 | 0.06 | -0.03 | 0.33 | 0.04 | 0.36 | 0.39 | -0.04 | 0.25 |
| G | -0.03 | -0.06 | -0.03 | -0.18 | -0.11 | 0.24 | -0.07 | -0.02 | 0.34 | -0.06 |
| U | -0.25 | -0.27 | -0.28 | -0.31 | -0.34 | -0.27 | -0.24 | -0.25 | -0.27 | -0.04 |

| nt position | -4 | -3 | -2 | -1 | 1 | 2 | 3 | 4 | 5 | 6 |
|---|---|---|---|---|---|---|---|---|---|---|
| A | -0.21 | **0.78** | **0.53** | 0.23 | **1.71** | -11.76 | -11.76 | -0.32 | -0.07 | **-0.79** |
| C | **0.78** | **-1.03** | 0.22 | 0.39 | -11.76 | -11.76 | -11.76 | -0.22 | **0.60** | **0.50** |
| G | -0.23 | 0.28 | **-0.65** | 0.00 | -11.76 | -11.76 | **2.19** | **0.70** | -0.40 | 0.40 |
| U | **-0.64** | **-1.37** | **-0.67** | **-0.99** | -11.76 | **2.01** | -11.76 | -0.29 | -0.32 | -0.23 |

| nt position | 7 | 8 | 9 | 10 | 11 | 12 | 13 | 14 | 15 | 16 |
|---|---|---|---|---|---|---|---|---|---|---|
| A | 0.05 | 0.07 | **-0.72** | 0.03 | -0.02 | -0.49 | 0.08 | 0.09 | **-0.68** | -0.02 |
| C | -0.06 | 0.23 | **0.57** | 0.03 | 0.20 | **0.50** | 0.08 | 0.07 | 0.30 | 0.05 |
| G | 0.37 | -0.27 | 0.45 | 0.27 | -0.37 | 0.26 | 0.28 | -0.17 | **0.62** | 0.40 |
| U | -0.44 | -0.10 | -0.47 | -0.39 | 0.11 | -0.31 | **-0.55** | -0.04 | -0.38 | **-0.51** |

| nt position | 17 | 18 | 19 | 20 |
|---|---|---|---|---|
| A | 0.05 | **-0.67** | 0.04 | -0.03 |
| C | -0.02 | 0.41 | 0.08 | 0.06 |
| G | -0.13 | 0.46 | 0.34 | -0.27 |
| U | 0.06 | -0.26 | **-0.55** | 0.18 |

## S.4 Drosophila cDNA relational database used for analysis

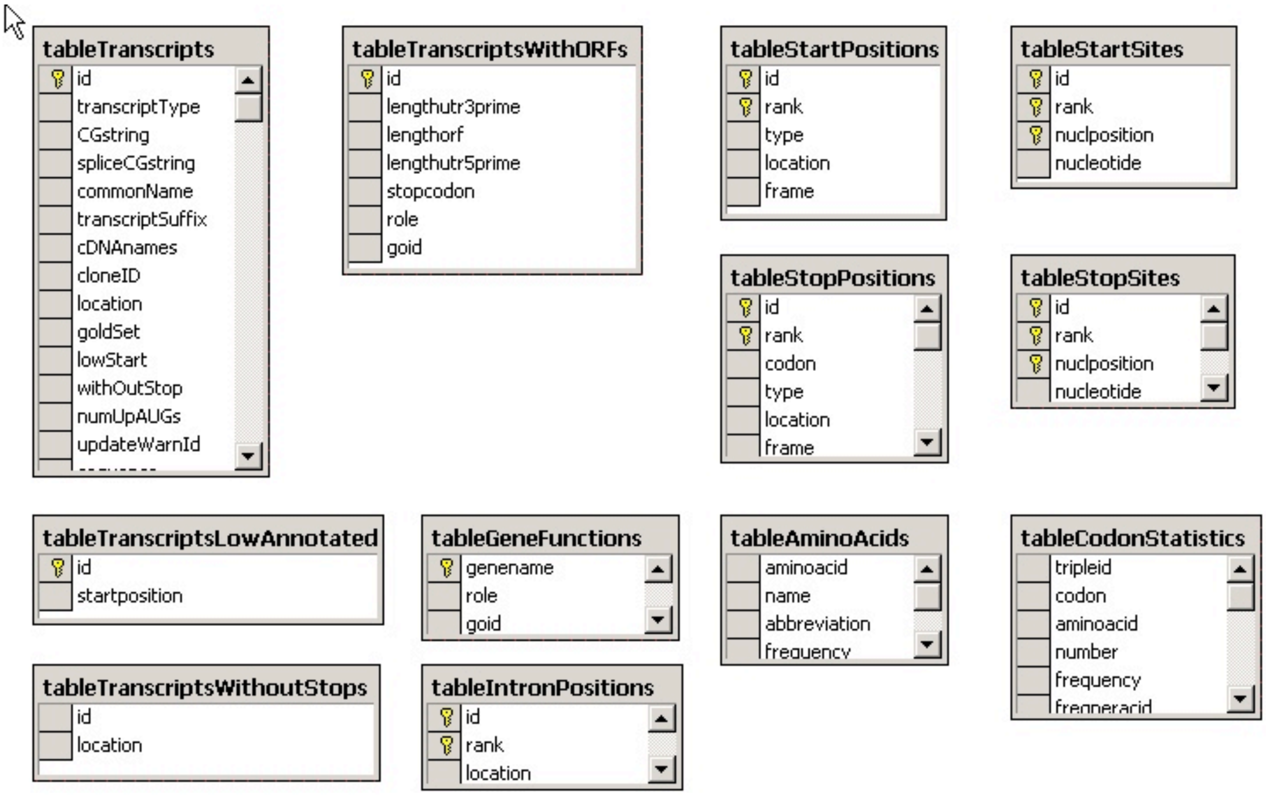


**Supplemental Fig. 4.** Schema of database used in analysis.

## S.5 Sequence sets

**Supplemental Table 5.** Summary of sequence sets used in this study.

| **Sequence set** | **Description** |
|---|---|
| Full cDNA set | 8,607 BDGP cDNAs (copies of mRNAs) analyzed in this study |
| $U_{200}$ | A subset of 3,470 cDNAs with annotated 5'UTR ≥ 200 nt |
| $S_{200}$ | 446 cDNAs with no upAUGs in their 5'UTRs of length ≥ 200 |
| $S_{100\text{-}199}$ | 1,004 cDNAs with no upAUGs in their 5'UTRs of length 100-199 |
| $S_{rand}$ | 5000 sequences with AUG flanked by random sequences conforming to background nt frequencies of the full cDNA set |
| BDGP predicted genes and transcripts | Full set of Drosophila genes and transcripts based on gene prediction algorithms and manual curation |
| BDGP Gold set cDNAs | Subset of BDGP cDNAs whose sequences match *Release 3* predicted BDGP transcripts |

**S.6 Analysis in *Saccharomyces cerevisiae***

We investigated the use of different reference sets for weight matrices in budding yeast by examining the correlations between gene expression and TRII scores.

Protein expression has been examined in yeast on a genomic scale through western analysis of epitope-tagged proteins (Ghaemmaghami et al. 2003). Ribosome densities for most transcripts have also been examined through deep sequence analysis (Ingolia et al. 2009); the ribosome densities correlate with protein expression levels.

Using a set of 1735 yeast genes with high-confidence annotation of their TISs (genes with cDNAs with 5'UTRs ≥ 100 and no upstream AUGs; Robbins-Pianka *et al.* submitted), we analyzed a subset of 138 genes with the highest protein expression per cell (top 10% of genes with detectable expression; Suppl. Fig. 5B). Similarly we analyzed 169 genes with the top 10% of ribosome densities (Suppl. Fig. 5C).

We constructed three weight matrices using the following reference sets:

- $w_{0up}$: 0-upAUG reference set of 1735 high-confidence TISs
- $w_{rand}$: random reference set of 1116 genes selected randomly from the full genome
- $w_{up50}$: upAUG reference set of 1475 genes with an upAUG within 50 nt of the annotated AUG start codon

The $w_{0up}$ weight matrix generally had weights of greater magnitude than the $w_{rand}$ or $w_{up50}$ weight matrices (Suppl. Fig. 5A; weights with absolute values ≥ 0.5 marked in bold).

The $w_{0up}$ weight matrix gave a stronger correlation between TRII score and log(protein expression) than the $w_{rand}$ or $w_{up50}$ weight matrices (Suppl. Fig. 5B). Similar results were observed when TRII score was compared with log(ribosome density) (Suppl. Fig. 5C). For both protein expression and ribosome density, the correlations with TRII score computed using $w_{0up}$ and $w_{up50}$ were significantly different according to the $Z_1$* statistic for comparing two dependent correlations ($p < 0.01$; Steiger 1980).

These trends towards higher correlation coefficients with $w_{0up}$ suggest that the TRII scores computed using the 0-upAUG reference set reflect better the translation potential of yeast genes. The distributions in Supplemental Figs. 5B and 5C suggest that higher TRII scores potentiate and may be required for high gene expression but some TISs with high TRII scores did not exhibit high expression in the conditions tested.

The TRII scores in Supplemental Fig. 5 were calculated using nucleotide positions -20 to 20. If nucleotide positions -40 to 40 were used instead, the correlations between protein expression or ribosome densities and TRII score were both more pronounced (Suppl. Fig. 6).

## A Weight Matrices

| nt position | -5 | -4 | -3 | -2 | -1 | 1 | 2 | 3 | 4 | 5 | Weight matrix |
|---|---|---|---|---|---|---|---|---|---|---|---|
| A | 0.22 | **0.53** | **1.02** | 0.47 | **0.58** | **1.63** | -10.76 | -10.76 | -0.11 | **-0.60** | |
| C | 0.15 | 0.16 | **-1.16** | 0.16 | -0.12 | -10.76 | -10.76 | -10.76 | -0.47 | **1.26** | $w_{0up}$ |
| G | -0.18 | -0.30 | 0.20 | **-0.52** | -0.15 | -10.76 | -10.76 | **2.62** | **0.77** | -0.11 | |
| U | -0.26 | **-0.74** | **-2.10** | -0.49 | **-0.71** | -10.76 | **1.58** | -10.76 | -0.18 | **-0.75** | |

| nt position | -5 | -4 | -3 | -2 | -1 | 1 | 2 | 3 | 4 | 5 | Weight matrix |
|---|---|---|---|---|---|---|---|---|---|---|---|
| A | 0.15 | 0.37 | **0.84** | 0.27 | 0.45 | **1.63** | -10.13 | -7.28 | -0.10 | -0.15 | |
| C | 0.10 | 0.09 | **-0.76** | 0.19 | -0.02 | -10.13 | -10.13 | -10.13 | -0.34 | **0.95** | $w_{rand}$ |
| G | 0.02 | -0.04 | 0.10 | -0.08 | -0.06 | -10.13 | -10.13 | **2.62** | **0.89** | -0.11 | |
| U | -0.24 | **-0.52** | **-1.25** | -0.41 | **-0.57** | -10.13 | **1.58** | -10.13 | -0.39 | **-0.70** | |

| nt position | -5 | -4 | -3 | -2 | -1 | 1 | 2 | 3 | 4 | 5 | Weight matrix |
|---|---|---|---|---|---|---|---|---|---|---|---|
| A | 0.09 | 0.28 | **0.50** | 0.20 | 0.28 | **1.63** | -10.53 | -10.53 | 0.15 | -0.11 | |
| C | 0.07 | 0.00 | **-0.62** | 0.02 | 0.00 | -10.53 | -10.53 | -10.53 | -0.34 | **0.72** | $w_{up50}$ |
| G | 0.28 | 0.10 | 0.37 | 0.24 | 0.14 | -10.53 | -10.53 | **2.62** | **0.60** | -0.02 | |
| U | -0.32 | -0.41 | **-0.64** | -0.41 | -0.43 | -10.53 | **1.58** | -10.53 | -0.40 | -0.48 | |

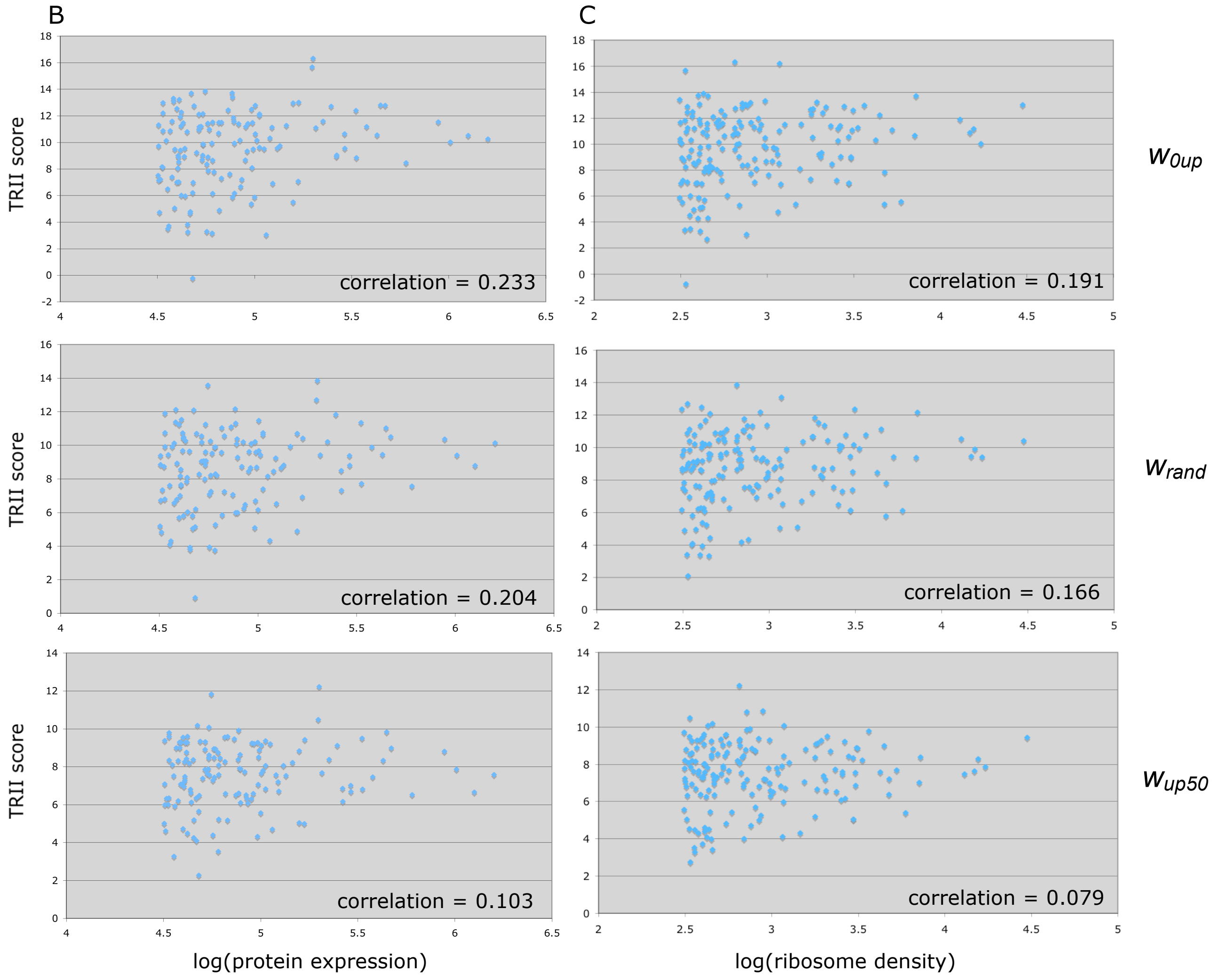


**Supplemental Fig. 5. Choice of Weight Matrices in Budding Yeast.** Weight matrices constructed with 0-upAUG reference sets ($w_{0up}$) gave a stronger correlation between TRII score and log(protein expression) or log(ribosome density) than weight matrices from randomly selected genes ($w_{rand}$) or genes with upAUGs within 50 nt of the annAUG ($w_{up50}$). Weight matrices in A show nt positions -5 to 5; weight matrices used in B and C were computed for nt positions -20 to 20. Protein expression was measured as protein molecules per cell; ribosome density was measured as ribosome tags that map to the first 200 nt of the annotated ORF. Panel B shows 138 genes with the highest protein expression; panel C shows 169 genes with the highest ribosome densities. Note that the correlation between TRII score and log(protein expression) is 0.224 (instead of when 0.233) if the 138 test sequences of genes with high protein expression are excluded when $w_{0up}$ is computed.

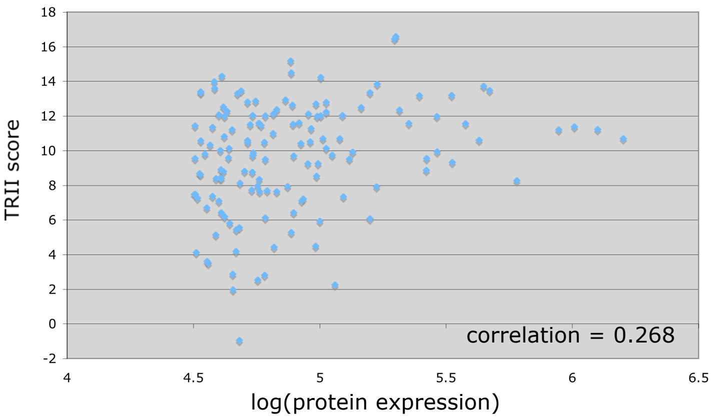


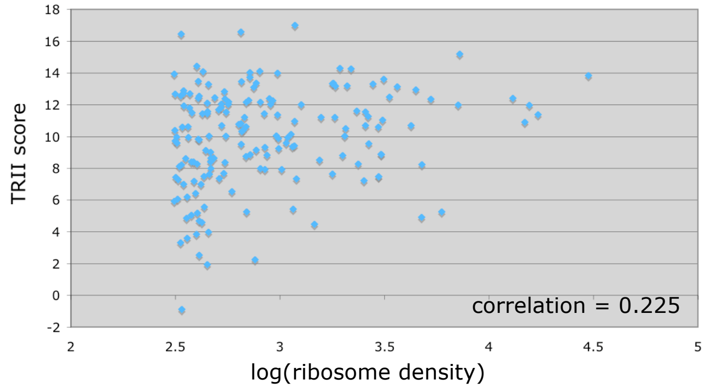


**Supplemental Fig. 6. Choice of Weight Matrix Window.**
TRII scores computed from nucleotide positions -40 to 40 show a stronger correlation with protein expression or ribosome density (compare with Suppl. Fig. 5). The $w_{0up}$ reference set was used for the weight matrix.
